# MicroRNA expression profiling of male breast cancer

**DOI:** 10.1186/bcr2348

**Published:** 2009-08-10

**Authors:** Matteo Fassan, Raffaele Baffa, Juan P Palazzo, Joshua Lloyd, Marco Crosariol, Chang-Gong Liu, Stefano Volinia, Hannes Alder, Massimo Rugge, Carlo M Croce, Anne Rosenberg

**Affiliations:** 1 Department of Urology, Thomas Jefferson University – Kimmel Cancer Center, 1112 College Building, 1025 Walnut Street, PA 19107, USA; 2 Department of Medical Diagnostic Sciences & Special Therapies – II Pathology Unit, University of Padova, via Gabelli 61, Padova 35121, Italy; 3 Department of Pathology, Thomas Jefferson University – Kimmel Cancer Center, 279 Jefferson Alumni Hall, 1020 Locust Street, PA 19107, USA; 4 Comprehensive Cancer Center, Ohio State University, 400 West 12th Avenue, Columbus, OH 43210, USA; 5 Department of Surgery, Thomas Jefferson University – Kimmel Cancer Center, 620 Curtis Building, 1015 Walnut Street, PA 19107, USA; 6 Present address: Medimmune, One Medimmune Way, Gaithersburg, MD 20878, USA

## Abstract

**Introduction:**

MicroRNAs (miRNAs) are a class of small noncoding RNAs that control gene expression by targeting mRNAs and triggering either translation repression or RNA degradation. Their aberrant expression may be involved in human diseases, including cancer. To test the hypothesis that there is a specific miRNA expression signature which characterizes male breast cancers, we performed miRNA microarray analysis in a series of male breast cancers and compared them with cases of male gynecomastia and female breast cancers.

**Methods:**

Paraffin blocks were obtained at the Department of Pathology of Thomas Jefferson University from 28 male patients including 23 breast cancers and five cases of male gynecomastia, and from 10 female ductal breast carcinomas. The RNA harvested was hybridized to miRNA microarrays (~1,100 miRNA probes, including 326 human and 249 mouse miRNA genes, spotted in duplicate). To further support the microarray data, an immunohistochemical analysis for two specific miRNA gene targets (*HOXD10 *and *VEGF*) was performed in a small series of male breast carcinoma and gynecomastia samples.

**Results:**

We identified a male breast cancer miRNA signature composed of a large portion of underexpressed miRNAs. In particular, 17 miRNAs with increased expression and 26 miRNAs with decreased expression were identified in male breast cancer compared with gynecomastia. Among these miRNAs, some had well-characterized cancer development association and some showed a deregulation in cancer specimens similar to the one previously observed in the published signatures of female breast cancer. Comparing male with female breast cancer miRNA expression signatures, 17 significantly deregulated miRNAs were observed (four overexpressed and 13 underexpressed in male breast cancers). The *HOXD10 *and *VEGF *gene immunohistochemical expression significantly follows the corresponding miRNA deregulation.

**Conclusions:**

Our results suggest that specific miRNAs may be directly involved in male breast cancer development and that they may represent a novel diagnostic tool in the characterization of specific cancer gene targets.

## Introduction

Breast cancer is a rare disease in men, representing less than 1% of all malignancies and responsible for 0.1% of male cancer deaths [1,2]. Despite its infrequent prevalence, male breast cancer can cause significant morbidity and mortality. In fact, it has a more aggressive clinical behavior compared with female breast cancer [2]. Most of the current knowledge regarding male breast cancer biology, natural history and treatment strategies is still limited and has been extrapolated from its female counterpart.

Genome-wide microarray gene expression analysis has been largely used to characterize human cancers. This approach allowed the identification of genes important in tumorigenesis. Microarray tools have been recently enriched by the development of platforms for the analysis of microRNA (miRNA) expression [3,4]. miRNAs are ~22-nucleotide small noncoding RNAs that modulate gene expression by binding to target mRNA by imperfect complementary, causing either mRNA degradation or translation inhibition [5]. In humans, aberrant expression of miRNAs contributes to carcinogenesis by promoting the expression of proto-oncogenes or by inhibiting the expression of tumor suppressor genes. Such oncomirs have been demonstrated in a variety of hematologic and solid malignancies [6-8], and also in female breast cancer [9-31]. Furthermore, interfering with miRNA expression, an altered experimental tumorigenesis has been observed [14,32].

Indeed, genome-wide profiling integrated by functional studies that involve overexpression and downregulation of miRNAs represents the current approach that is most likely to yield advances in the new field of noncoding RNA research. Moreover, miRNAs are, in contrast to most mRNAs, long-lived *in vivo *and very stable *in vitro*, which might be critical in a clinical setting and may allow analysis of paraffin-embedded samples [11].

Previous studies have demonstrated that there is a large number of deregulated miRNAs in human breast cancer (in particular, miR-10b, miR-17-5p, miR-21, miR-27a, miR-27b, miR-125a, miR-125b, miR-126, miR-145, miR-155, miR-200c, miR-206, miR-336 and the let-7 family) [9-31]. The miRNA signatures have been correlated with clinicopathological and prognostic parameters such as tumor size, nodal involvement, vascular invasiveness, ErbB2, estrogen receptor status and chemotherapy resistance [9-11,13,21-24,27,28,30,31]. Moreover, a functional polymorphism in the miR-146a gene has been correlated with the predisposition to an earlier age of onset of familial breast and ovarian cancers [26]. Three recent studies proved the involvement of miRNAs in breast cancer metastases [14,32,33]. For instance, miR-10b overexpression leads to tumor invasion and metastasis by suppressing *HOXD10 *and indirectly activating the prometastatic gene *RHOC *[14]. Two other miRNAs (miR-373 and miR-520c) can also promote tumor invasion and metastasis, at least in part by regulating the *CD44 *gene [32]. In addition, miR-335, miR-206 and miR-126 have been identified as suppressors of breast cancer metastasis [33].

These findings strongly suggest that miRNA expression profiles could represent a promising new class of cancer biomarkers. Moreover, in the future, miRNAs could be potentially used as innovative and targeted therapeutics [34].

In the present report we analyze the genome-wide miRNA expression profile of 23 male breast carcinoma cases versus five male gynecomastia cases, and of 10 female breast carcinoma samples. Our data indicate a miRNA signature associated with male breast carcinoma and suggest miRNA deregulation as an important event in male breast cell transformation.

## Materials and methods

### Tissue samples

After exempt status for the study was granted by the Institutional Review Board of Thomas Jefferson University, specimens from 28 male patients, including 23 cases of breast cancer and five cases of gynecomastia, and from 10 female patients affected by ductal breast carcinoma were identified from the archival files of the Department of Pathology of Thomas Jefferson University (Philadelphia, PA, USA). The patients had undergone treatment by either simple mastectomy or wide local excision and radiotherapy. Adjuvant therapy was not given. All cases were reviewed by two pathologists (JPP and RB) and the diagnoses confirmed. Four 15 μm sections were obtained from each case.

For the immunohistochemical study, formalin-fixed and paraffin-embedded tissues of 10 cases of male ductal breast carcinoma and five cases of gynecomastia were obtained from the archival files of the Department of Surgical Pathology of the University of Padova.

All patients considered in this study gave their written informed consent. The male breast cancer samples have been considered in an earlier publication [35].

### MicroRNA microarray

Tissue sections were deparaffinized with xylene at 50°C for 3 minutes. Total RNA extraction was undertaken using the RecoverAll kit (Ambion Inc, Austin, TX, USA) according to the manufacturer's instructions. RNA labeling and hybridization on miRNA microarray chips were performed as previously described [36]. Briefly, 5 μg total RNA from each sample were reverse-transcribed using biotin end-labeled random-octamer oligonucleotide primer. Hybridization of biotin-labeled complementary DNA was performed on a new Ohio State University custom miRNA microarray chip (OSU_CCC version 4.0), which contains ~1,100 miRNA probes – including 326 human and 249 mouse miRNA genes, spotted in duplicate [3]. The hybridized chips were washed and processed to detect biotin-containing transcripts by streptavidin- Alexa647 conjugate and were scanned on an Axon 4000B microarray scanner (Axon Instruments, Sunnyvale, CA, USA).

### Statistical and bioinformatics analysis

Microarray images were analyzed using GENEPIX PRO 6.0 (Axon Instruments). Average values of the replicate spots of each miRNA were background subtracted, normalized and further analyzed. Normalization was performed using quantiles [3]. The microarray data have been deposited at the National Center for Biotechnology Information Gene Expression Omnibus repository [GEO:GSE17155].

miRNAs that are differentially expressed between breast cancer and gynecomastia were identified using a random-variance *t *test. The random-variance *t *test is an improvement over the standard separate *t *test as it permits sharing information among genes about within-class variation without assuming that all genes have the same variance [37]. Genes were considered statistically significant if their *P *value was less than 0.01. A stringent significance threshold was used to limit the number of false positive findings [38]. Only mature miRNAs that are differentially expressed are reported.

Our samples were not microdissected so, to further avoid the influence of heterogeneity/a different percentage of cell types between compared samples (cancer cells versus surrounding stromal tissue cells), we performed an enrichment analysis for the gynecomastia versus male breast cancer groups. The enrichment analysis was performed by enriching the original microarray analysis data for tumor area. For this purpose, the tumor area – defined as the percentage of tumor over the stromal counterpart – was histologically determined by two pathologists (JPP and RB).

### Quantitative real-time PCR

The single-tube TaqMan miRNA Assay (Applied Biosystems, Foster City, CA, USA) was used to detect and quantify mature miRNAs on Applied Biosystems real-time PCR instruments in accordance with the manufacturer's instructions. Normalization was performed with the small nuclear RNA U48 (RNU48; Applied Biosystems). All real-time reactions, including no-template controls and real-time minus controls, were run in a GeneAmp PCR 9700 thermocycler (Applied Biosystems). Gene expression levels were quantified using the ABI Prism 7900HT Sequence Detection System (Applied Biosystems).

Comparative real-time PCR was performed in triplicate, including no-template controls. The fold difference for each sample was obtained using the expression 2-^ΔCt^, in which Ct is the threshold cycle (the threshold cycle is the cycle number at which the fluorescence generated within a reaction crosses the threshold) and ΔCt is the difference between the average Ct value of a sample gene and the average Ct for the endogenous reference RNU48. Total RNA from 19 of the 23 male breast cancers and from the five gynecomastia samples were used in the quantitative real-time PCR analysis. The RNA was extracted from different tissue blocks to those used in the miRNA microarray study.

### Immunohistochemistry

Staining was performed automatically (Ventana Benchmark XT system; Ventana Medical Systems, Touchstone, AZ, USA) for vascular endothelial growth factor (VEGF) (catalog number sc-152 – VEGF-A20, 1:100; Santa Cruz Biotechnology Inc., Santa Cruz, CA, USA) and homeobox-D10 (HOXD10) (catalog number sc-66926, 1:50; Santa Cruz Biotechnology Inc.) according to the manufacturer's instructions. Sections were then lightly counterstained with hematoxylin. Appropriate positive and negative controls were run concurrently. The expression of each immunohistochemical (IHC) marker was jointly scored by two pathologists (MF and MR). Cytoplasmic positivity was considered for VEGF, and both cytoplasmic and membranous positivity were considered for HOXD10. Regarding the IHC score intensity, positive staining was semi-quantified with a three-tier system: negative positivity, low-grade positivity, high-grade positivity. Results were reported as positive when staining was observed in at least 5% of the tumor cells. When the staining intensity was heterogeneous, the highest result was retained for scoring.

## Results

### MicroRNA expression signatures discriminate between gynecomastia and cancer and between male and female breast cancers

To identify deregulated miRNAs in male breast cancers, the miRNA expression profiles were determined for five male gynecomastia samples, 23 male breast cancer samples and 10 female breast cancer samples using a custom microarray platform proven to give robust results, as validated by several studies [3,4,10,12]. All of the 23 male breast cancer samples pathologically corresponded to invasive ductal breast carcinoma. The clinicopathological characteristics are summarized in Table 1.

**Table 1:** Clinicopathological characteristics of the 23 male breast cancer samples

Characteristic	Value
Age	71.2 ± 9.0 (72.0)
Tumor size	20.0 ± 9.6 (20.0)
Diagnosis	
Invasive ductal carcinoma	11 (47.8%)
Invasive ductal carcinoma + ductal carcinoma *in situ*	12 (52.2%)
Nuclear grade	
I	3 (13.0%)
II	11 (47.8%)
III	9 (39.1%)
Histological grade	
I	2 (8.7%)
II	12 (52.2%)
III	9 (39.1%)
Cytokeratin 5/6	
0	19 (82.6%)
+	1 (4.3%)
++	3 (13.0%)
+++	0 (0.0%)
Cytokeratin 14	
0	19 (82.6%)
+	2 (8.7%)
++	0 (0.0%)
+++	2 (8.7%)
Cytokeratin 18	
0	1 (4.3%)
+	0 (0.0%)
++	3 (13.0%)
+++	19 (82.6%)
pT	
1	13 (56.5%)
2	10 (43.5%)
pN	
0	8 (34.8%)
1	8 (34.8%)
2	1 (4.3%)
*x*	6 (26.1%)
pM	
0	8 (34.8%)
1	2 (8.7%)
*x*	13 (56.5%)
Estrogen receptors	
Positive	12 (95.7%)
Negative	1 (4.3%)
Not available	10
Progesterone receptors	
Positive	13 (100.0%)
Negative	0 (0.0%)
Not available	10

Data presented as the mean ± standard deviation (median) or as *n *(%). -, negative; +, < 25%; ++, 25 to 50%; and +++, > 50%.

Comparison analysis showed the differential expression of several miRNA genes between gynecomastia and male breast cancer. In detail, we identified 17 miRNAs with increased expression and 26 miRNAs with decreased expression in male breast cancer samples in comparison with gynecomastia samples (Table 2).

**Table 2:** MicroRNA differential expression between male breast cancers and cases of male gynecomastia

MicroRNA	Cancer normalized	Gynecomastia normalized	Fold change^a^	*P *value
hsa-miR-26a-2	82.5	15.4	5.357	0.002517
hsa-miR-26b	137.7	27.2	5.062	0.006413
hsa-miR-499-3p	290.6	63.9	4.548	0.005332
hsa-miR-607	389.4	87	4.476	0.001533
hsa-miR-135b	199.3	45.7	4.361	0.008074
hsa-miR-616	269.9	66	4.089	0.008253
hsa-miR-769-5p	184.4	53.6	3.44	0.006534
hsa-miR-330-5p	291.1	89.2	3.263	0.001353
hsa-miR-132	155.7	49.2	3.165	0.007551
hsa-miR-149	461.7	161.6	2.857	0.003765
hsa-miR-557	686.1	286.4	2.396	0.005408
hsa-miR-29b-2	1,273.7	604.9	2.106	1.12 × 10^-5^
hsa-miR-657	2,499.3	1,277.2	1.957	0.000899
hsa-miR-483-3p	18,985.9	10,525.1	1.804	0.001411
hsa-miR-371-3p	9,741.4	5,642.1	1.727	0.003106
hsa-miR-593	1,805.1	1,051.3	1.717	0.001461
hsa-miR-596	1,430.8	909.5	1.573	0.000370
hsa-miR-92a	6,490.7	11,614.8	- 1.789	0.008752
hsa-miR-145	1,561.5	3,649.3	- 2.336	0.000110
hsa-miR-99b	678.8	1,697.8	- 2.500	0.000985
hsa-miR-214	1,392.7	3,717.6	- 2.667	4.31 × 10^-5^
hsa-miR-191	1,337.8	4,112	- 3.077	0.003671
hsa-miR-454	11.2	36.2	- 3.236	0.006250
hsa-miR-10a	1,765.9	5,748.8	- 3.257	5 × 10^-6^
hsa-miR-195	188.2	865.9	- 4.608	0.008269
hsa-miR-10b	13.4	84.9	- 6.329	0.000451
hsa-miR-130a	451.8	2,868.5	- 6.329	0.003837
hsa-miR-374a	18.1	128.8	- 7.092	0.000556
hsa-miR-146b-5p	190.4	1,459.9	- 7.692	0.005392
hsa-miR-146a	129.2	1,070.7	- 8.264	0.007859
hsa-miR-181c	63.9	539.1	- 8.403	0.001424
hsa-miR-218	10	99.7	- 10.000	3 × 10^-7^
hsa-let-7g	59	611.5	- 10.417	0.002014
hsa-miR-15b	21.5	229.2	- 10.638	0.001555
hsa-miR-125a-5p	62.5	790.1	- 12.658	0.001136
hsa-miR-223	79.8	1,158.8	- 14.493	0.000837
hsa-miR-99a	70	1,111.4	- 15.873	0.000261
hsa-miR-140-3p	18.1	291.8	- 16.129	2.31 × 10^-5^
hsa-miR-126	16.7	2,71.9	- 16.393	5.67 × 10^-5^
hsa-miR-199b-3p	136	2,517.8	- 18.519	0.003903
hsa-miR-100	84.9	1,757.3	- 20.833	0.000104
hsa-miR-199a-5p	35.3	782.6	- 22.222	0.000256
hsa-miR-125b	95.3	2,992.6	- 31.250	9.91 × 10^-5^

^a^Presented as the actual change in expression.

Although our samples were partially microdissected, contamination by surrounding stromal cells was unavoidable. We therefore performed an enriched analysis for the tumor area (Table 3). miR-499-3p and miR-330-5p miRNA follow the enrichment, decreasing in the gynecomastia and increasing in the male breast cancer samples.

**Table 3:** MicroRNA differential expression between male breast cancers and male gynecomastia after enrichment for tumor cellularity

MicroRNA	Cancer normalized	Gynecomastia normalized	Fold change^a^	*P *value
hsa-miR-499-3p	300.2	17.7	16.96	0.000361
hsa-miR-129-3p	124.3	10.0	12.43	0.001482
hsa-miR-330-5p	322.1	66.6	4.836	0.003715
hsa-miR-10a	1829.8	6683.6	- 3.650	0.000353
hsa-miR-191	1655.3	6552.6	- 3.952	0.007160
hsa-miR-338-5p	17.3	106.7	- 6.173	0.005437
hsa-miR-218	10.0	74.6	- 7.463	0.000527
hsa-miR-369-3p	17.5	151.8	- 8.674	0.002524
hsa-miR-454	11.8	102.8	- 8.711	0.000753
hsa-miR-10b	15.1	132.8	- 8.772	0.005204
hsa-miR-374a	17.6	238.3	- 13.514	0.000447
hsa-miR-126	18.8	315.2	- 16.667	0.003277
hsa-miR-140-3p	18.6	340.7	- 18.182	0.000843

^a^Presented as the actual change in expression.

No significant association has been observed between clinicopathological tumor characteristics and miRNA gene expression.

**Figure 1:**
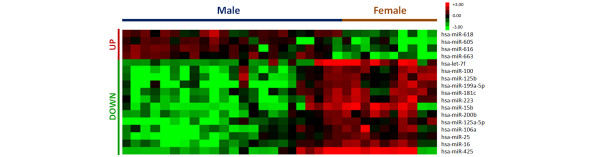
MicroRNA differential expression in male versus female breast cancers. Hierarchical clustering of the 17 microRNA genes with a significantly different expression. Rows, individual genes; columns, individual tissue samples. Pseudocolors indicate transcript levels below, equal to, or above the mean (green, black, and red, respectively). The scale represents the intensity of gene expression (log_2 _scale ranges between -3 and 3).

In the search for male/female breast cancer specific miRNAs, we correlated male breast cancer with female breast cancer miRNA gene expression profiles. This sex-specific comparison showed a different expression of 17 miRNAs between the two categories, with four upregulated and 13 downregulated miRNAs in male breast cancers (Table 4 and Figure 1).

**Table 4:** MicroRNA differential expression between male and female breast cancers

MicroRNA	Male normalized	Female normalized	Fold change^a^	*P *value
hsa-miR-663	492.9	77.5	6.369	7.1 × 10^-6^
hsa-miR-618	428.3	106.7	4.444	2.0 × 10^-7^
hsa-miR-605	174.0	44.4	3.922	0.000373
hsa-miR-616	309.2	86.0	3.597	0.000213
hsa-miR-200b	147.6	642.8	- 4.354	0.000144
hsa-miR-181c	145.9	656.1	- 4.500	0.000624
hsa-miR-106a	199.3	914.7	- 4.589	0.000594
hsa-miR-125a-5p	131.0	697.9	- 5.326	0.000698
hsa-miR-16	351.6	2021.7	- 5.750	0.000871
hsa-miR-25	309.3	1875.1	- 6.062	0.000992
hsa-miR-100	196.8	1206.9	- 6.132	0.000160
has-let-7f	39.0	241.4	- 6.186	0.000380
hsa-miR-125b	206.6	1427.7	- 6.912	0.000513
hsa-miR-15b	48.7	364.5	- 7.108	0.000508
hsa-miR-425	53.5	436.3	- 8.161	0.000614
hsa-miR-199a-5p	84.7	710.0	- 8.387	0.000306
hsa-miR-223	167.3	1414.6	- 8.454	0.000111

^a^Presented as the actual change in expression.

### Quantitative real-time PCR analysis

**Figure 2:**
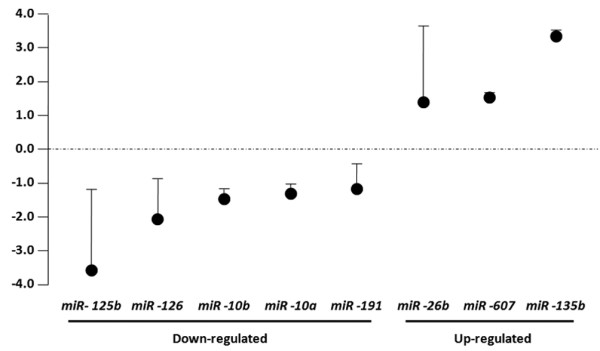
Quantitative real-time PCR validation of microRNA microarray results in male breast cancers. Relative expression of microRNAs in male breast cancer compared with cases of gynecomastia by real-time PCR. miR-125b, miR-126, miR-10b, miR-10a and miR-191 were underexpressed in cancer samples, whereas miR-26b, miR-607 and miR-135b were overexpressed. Dots, normalized ratio microRNA gene expression values (breast cancers/gynecomastia); error bars, standard deviation.

To confirm the results of microarray analysis, we performed quantitative real-time PCR analysis on a limited number of samples (19 cancer samples, five gynecomastia samples) using probes corresponding to miR-125b, miR-126, miR-10b, miR-10a, miR-191, miR-26b, miR-607 and miR-135b (Figure 2). The quantitative real-time PCR analysis confirmed the results obtained by microarray analysis. In fact, miR-125b, miR-126, miR-10b, miR-10a and miR-191 were underexpressed whereas miR-26b, miR-607 and miR-135b were overexpressed in cancer samples examined, in comparison with the gynecomastia samples.

### Immunohistochemical analysis

**Figure 3:**
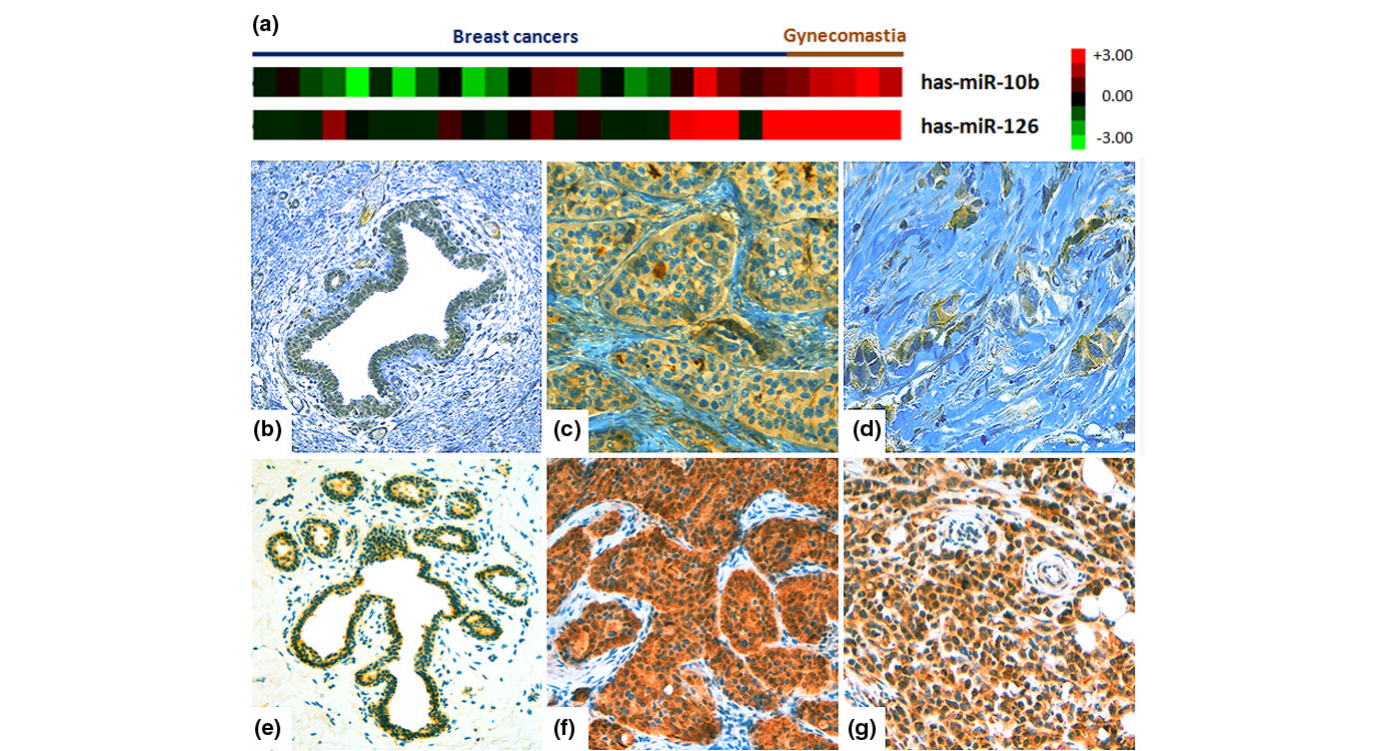
miR-10b and miR126 genes are downregulated in male breast cancers versus gynecomastia. **(a) **Columns represent individual tissue samples. Pseudocolors indicate transcript levels below, equal to, or above the mean (green, black, and red, respectively). The scale represents the intensity of gene expression (log_2 _scale ranges between -3 and 3). Representative examples of **(b), (c), (d) **homeobox-D10 and **(e), (f), (g) **vascular endothelial growth factor immunohistochemical expression in male breast cancers and gynecomastia samples. (b) and (e) Weak immunohistochemical staining in gynecomastia samples. (c), (d), (f) and (g) Positive immunoreactions in male breast cancer samples.

To further confirm our microarray data, and to demonstrate that miRNA analysis may be important in investigations into male breast cancer specific genes, we sought to correlate the expression of significantly deregulated miRNA genes in male breast cancer samples with two of their previously reported targets (Figure 3a). For this purpose, we performed an IHC analysis on the *HOXD10 *and *VEGF *genes [14,39].

The *HOXD10 *gene represses the expression of genes involved in cell migration and extracellular matrix remodeling in breast cancer cells, and it is regulated by miR-10b [14]. Since we observed miR-10b to be downregulated in male breast cancers, we therefore decided to analyze HOXD10 expression in formalin-fixed paraffin-embedded tissue samples obtained from 10 male breast cancer patients and five gynecomastia patients. As expected, the five gynecomastia samples showed a weak cytoplasmic HOXD10 IHC positivity, whereas 8/10 male breast cancers samples showed HOXD10 moderate- strong cytoplasmic positivity with membranous reinforcement (Figure 3b to 3d).

*VEGF *is a positive regulator of angiogenesis, and its expression is upregulated in many types of cancers, including breast cancers. VEGF is also a target protein of miR-126 [39]. Given the downregulation of miR-126 in male breast cancers, we tested VEGF IHC expression in our series of male breast cancers/gynecomastia. In gynecomastia samples VEGF showed weak cytoplasmic immunoreactions, whereas in 9/10 males a strong cytoplasmic reactivity was observed (Figure 3e to 3g).

## Discussion

Aberrant miRNA expression patterns have been described in a variety of hematologic and solid-organ malignancies, and several miRNA signatures have also been described for female breast cancer [10-30]. In the present study we have identified a global expression pattern of miRNAs that can differentiate gynecomastia from male breast cancer and can differentiate male breast cancer from female cancers.

Gynecomastia is the most common clinical and pathologic abnormality of the male breast. It results from hypertrophy of breast tissue. Numerous conditions have been associated with gynecomastia, but the pathophysiological bases are due to an imbalance of sex hormones and the tissue responsiveness to them.

Previous studies of miRNA expression in human breast cancer have focused on comparing normal tissues with tumor samples [10,11,21]. Because of the absence in men of a normal nonhypertrophic breast gland, we choose to compare gynecomastia with male breast cancer. In fact, gynecomastia has a higher amount of breast epithelium than normal breast. Gynecomastia could therefore be considered a good normal control for male breast cancer.

We identified a miRNA signature for male breast cancer, composed of 43 miRNAs that were differently expressed between tumors and gynecomastia samples. In particular, 17 miRNAs were upregulated and 26 miRNAs were downregulated in cancers (Table 2). Interestingly, in this signature we identified miRNAs that had previously been associated with breast cancers compared with normal tissues. Two overexpressed miRNAs – miR-149 [10] and miR-29b [12,21,22] – have already been described as upregulated in other female breast cancer studies. On the other hand, miR-145 [10,20], miR-10b [10], let-7g [19], miR-125a-5p [10,31], miR-125b [31] and miR-126 [40] have been described as downregulated.

According to previous reports in female breast carcinogenesis, the most interesting and promising miRNAs of this male breast cancer signature are miR-10b, miR-126, miR-125a-5p and miR-125b [14,31,40].

miR-10b has been previously described as downregulated in female breast cancer compared with normal tissue [10], but it has been also associated with the induction of tumor invasion and metastasis of breast cancer derived cells by targeting *HOXD10 *[14]. The downregulation of *HOXD10 *permits subsequently the expression of the prometastatic gene product *RHOC*, favoring, in turn, cancer cell migration and invasion [14]. Gee and colleagues, however, showed that miR-10b is not significantly associated with breast cancer metastasis in a large series of early-stage breast cancers [41].

We observed in our tumor samples a downregulation of miR-125a-5p, miR-125b, miR-126, miR-145 and let-7g genes, which have been shown to be related to hormonal settings and ErbB2 status of the tumor: miR-125a-5p and miR-125b downregulate ErbB2 and ErbB3 expression [31], miR-126 and let-7g are upregulated in ErbB2-negative tumors, whereas miR-145 is upregulated in ErbB2-negative tumors and upregulated in estrogen-receptor-positive and progesterone-receptor-positive tumors [13].

Our IHC study supports previous reports on the relationship of miR-10b and miR-126 and their respective gene targets [14,39]. Larger prospective studies are needed to confirm our IHC data, but we have further suggested that miRNA profiling may be helpful in the discovery of new breast cancer gene targets.

Although our tumor samples were partially microdissected, contamination with surrounding stromal tissue is inevitable. For this reason we performed an enrichment analysis of our data considering the amount of tumor tissue analyzed (Table 3). In this analysis, we observed that two miRNAs – miR-499-3p and miR-330-5p – were upregulated in cancer samples and followed the enrichment, suggesting they are key miRNAs in male breast cancer. Because these two miRNAs have not yet been associated with cancer development, we are planning to test their possible role in breast cell transformation with functional studies. Other miRNAs similarly altered after the enrichment, such as miR-191, miR-454, miR-10a, miR-374a, miR-10b, miR-218, miR-140-3p and miR-126, were downregulated in cancer. This group of miRNAs could also be important in the transformation of male breast cells. On the other hand, one could question whether these miRNAs are basically expressed in normal stromal cells and the enrichment is only a secondary effect to the loss of the stromal counterpart.

Of interest, from a histological point of view, male breast cancer is usually an invasive ductal carcinoma [35]. This histotype specificity seems one of the major differences between male and female breast cancer biology. We therefore analyzed the miRNA expression profiles of male/female breast cancer samples, looking for miRNAs differentially expressed between the two clinical categories. We identified 17 deregulated miRNAs (Table 4). Further studies considering large series of male/female breast tumors need to be conducted to address a possible role of miRNAs concerning the biological and prognostic tumor characteristics.

## Conclusions

The present report provides the first study on miRNA expression in male breast cancer. Considering gynecomastia as a potentially benign counterpart of male breast glands, we identified miRNAs that are differentially expressed between cancer and gynecomastia. Moreover, we investigated the differences between miRNA gene expression profiles in male versus female breast cancers.

The functional significance of miRNA dysregulation we have shown needs to be further investigated. Our results suggest that specific miRNAs may be directly involved in male breast cancer development and that they may represent a novel diagnostic tool in the characterization of specific cancer gene targets.

## Abbreviations

HOXD10: homeobox-D10; IHC: immunohistochemical; miRNA: microRNA; PCR: polymerase chain reaction; VEGF: vascular endothelial growth factor.

## Competing interests

The authors declare that they have no competing interests.

## Authors' contributions

All authors directly participated in the planning and execution of the study, and read and approved the final version of the manuscript.
